# The genome sequence of the channel bull blenny, *Cottoperca gobio *(Günther, 1861)

**DOI:** 10.12688/wellcomeopenres.16012.1

**Published:** 2020-06-24

**Authors:** Iliana Bista, Shane A. McCarthy, Jonathan Wood, Zemin Ning, H. William Detrich III, Thomas Desvignes, John Postlethwait, William Chow, Kerstin Howe, James Torrance, Michelle Smith, Karen Oliver, Vertebrate Genomes Project Consortium, Eric A. Miska, Richard Durbin

**Affiliations:** 1 Wellcome Sanger Institute, Cambridge, CB10 1SA, UK; 2 Department of Genetics, University of Cambridge, Cambridge, CB2 3EH, UK; 3 Department of Marine and Environmental Sciences, Northeastern University Marine Science Center, Massachusetts, MA 01908, USA; 4 Institute of Neuroscience, University of Oregon, Eugene, OR 97403-1254, USA; 5 Gurdon Institute, University of Cambridge, Cambridge, CB2 1QN, UK

**Keywords:** Cottoperca gobio, channel bull blenny, genome assembly chromosomal, Notothenioidei

## Abstract

We present a genome assembly for *Cottoperca gobio *(channel bull blenny, (Günther, 1861)); Chordata; Actinopterygii (ray-finned fishes), a temperate water outgroup for Antarctic Notothenioids. The size of the genome assembly is 609 megabases, with the majority of the assembly scaffolded into 24 chromosomal pseudomolecules. Gene annotation on Ensembl of this assembly has identified 21,662 coding genes.

## Species taxonomy

Eukaryota; Metazoa; Chordata; Vertebrata; Gnathostomata; Actinopterygii; Teleostei; Clupeocephala; Percomorphaceae; Perciformes; Notothenioidei; Bovichtidae; Cottoperca; *Cottoperca gobio* (Günther, 1861) - synonym: *Cottoperca trigloides* ( Balushkin, 2000), NCBI taxid: 56716.

## Background

*Cottoperca gobio* (channel bull blenny) is a member of the Bovichtidae family of the Notothenioidei, a fish group endemic to the Southern Ocean. The Bovichtidae (thornfishes), are considered to be the most basally diverging family of notothenioids and are less adapted to life in the extreme cold in comparison to Antarctic members of the clade ( Near *et al*., 2015). *C. gobio* occupies the Patagonian regions of Chile and Argentina, and the area around the Falkland Islands. In contrast to Antarctic notothenioids (cryonotothenioids), the Bovichtidae do not produce antifreeze glycoproteins (AFGPs), a key adaptation to extreme Antarctic cold ( Chen *et al*., 1997; Cheng *et al*., 2003) and their hemoglobins possess slightly higher oxygen affinity than most high-Antarctic species ( Giordano *et al*., 2006; Giordano *et al*., 2009). Cytogenetic investigation of *C. gobio* showed that the karyotype of this species consists of 2n=48 chromosomes ( Pisano *et al*., 1995). This condition, shared by other Bovichtidae, is considered to be the ancestral karyotype condition for all notothenioids ( Mazzei *et al*., 2006). 

Here, we present a chromosomally complete genome sequence of *Cottoperca gobio* generated using specimens collected south of the Falkland Islands/Islas Malvinas. We trust that this genome sequence will be used to aid analysis of population structure and phylogeography of non-Antarctic and Antarctic notothenioid fish species, which are increasingly under threat due to climate change and human activities ( Dornburg *et al*., 2017).

## Genome sequence report

The *C. gobio* genome was sequenced from a specimen collected under permits to fish in territorial waters of the Falkland Islands/Islas Malvinas issued by the United Kingdom, by the Falkland Islands Government, and by Argentina. The genome assembly for *C. gobio* (fCotGob3.1) is based on a combination of data from four technologies, including 75x coverage Pacific Biosciences (PacBio) single-molecule long reads (N50 14 kb), 54x coverage of Illumina data generated from a 10X Genomics Chromium library (estimated molecule length N50 43 kb), and BioNano Saphyr two-enzyme data (BspQI and BssSI). Additionally, 145x coverage of Illumina HiSeqX data were obtained from a Hi-C library prepared by Arima Genomics using tissue from a second individual (fCotGob2, spleen tissue).

**Figure 1.**
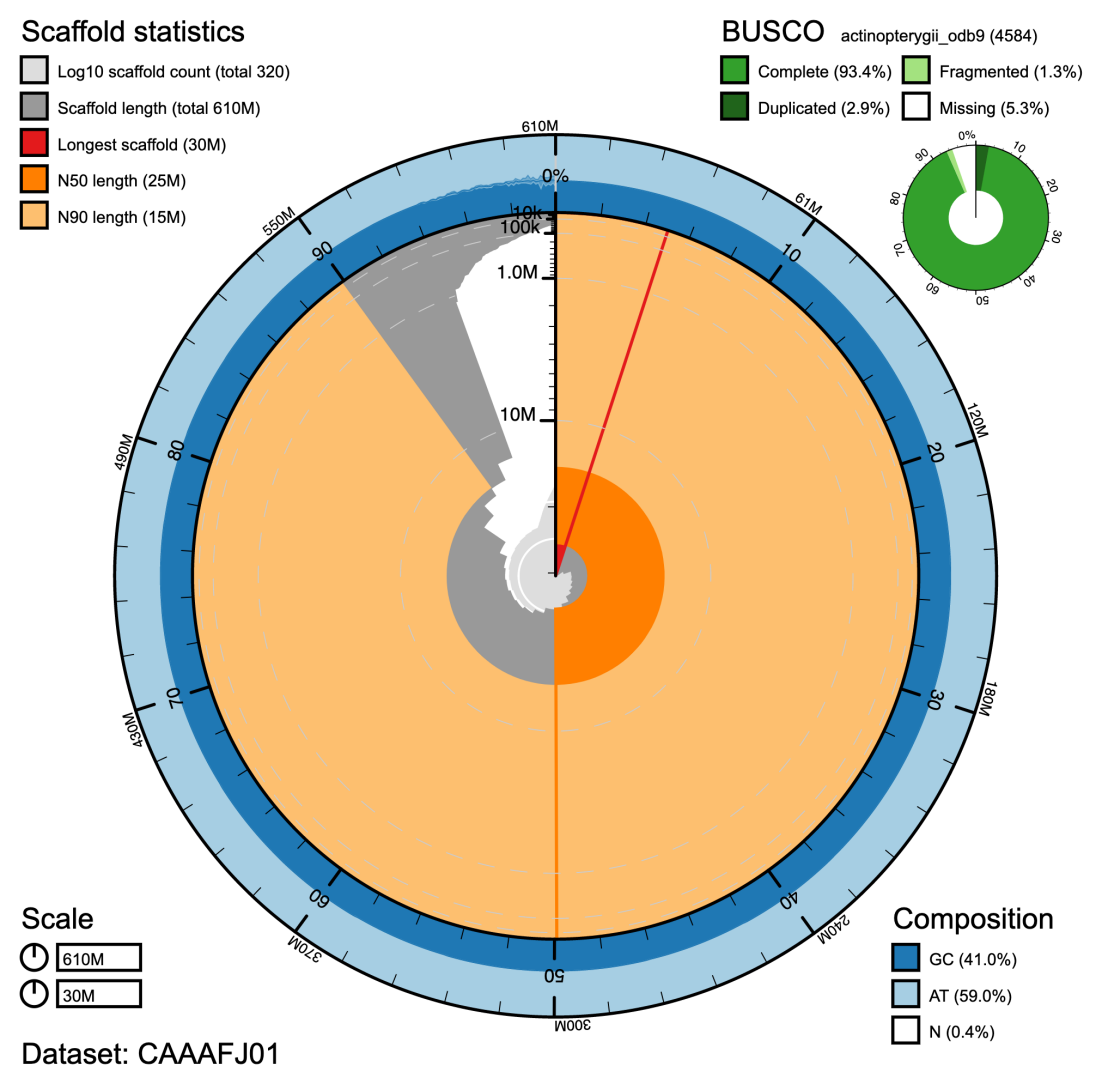
Genome assembly of Cottoperca gobio, fCotGob3.1. - BlobToolKit Snailplot, showing N50 metrics and BUSCO gene completeness. BlobToolKit plots are available at: fCotGob3.1 - BlobToolKit.

**Figure 2.**
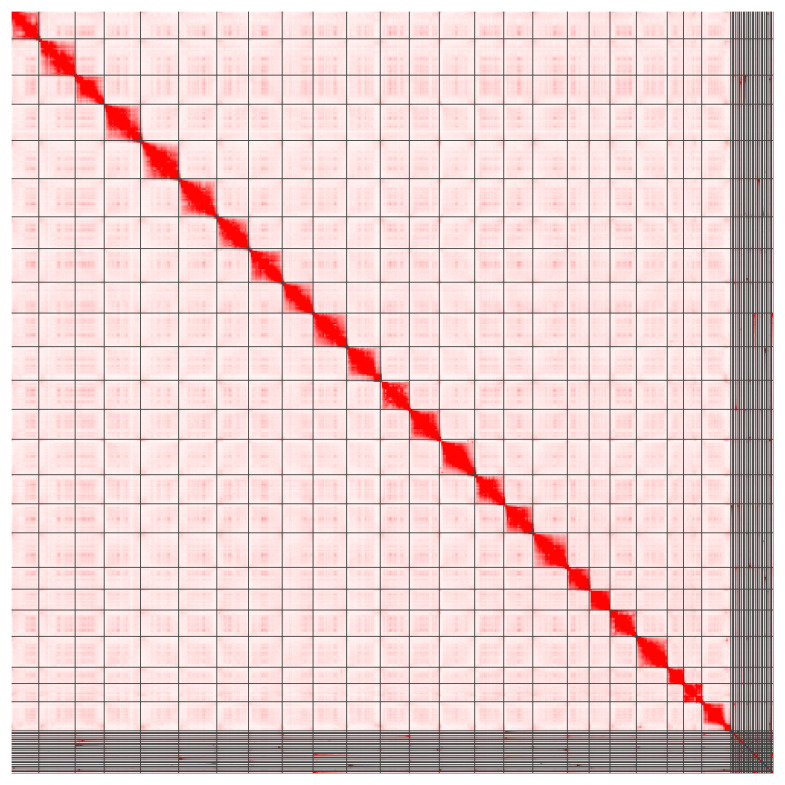
Hi-C contact map for the genome assembly of *Cottoperca gobio*, fCotGob3.1. Visualized in Juicebox ( Durand *et al.,* 2016).

**Figure 3.**
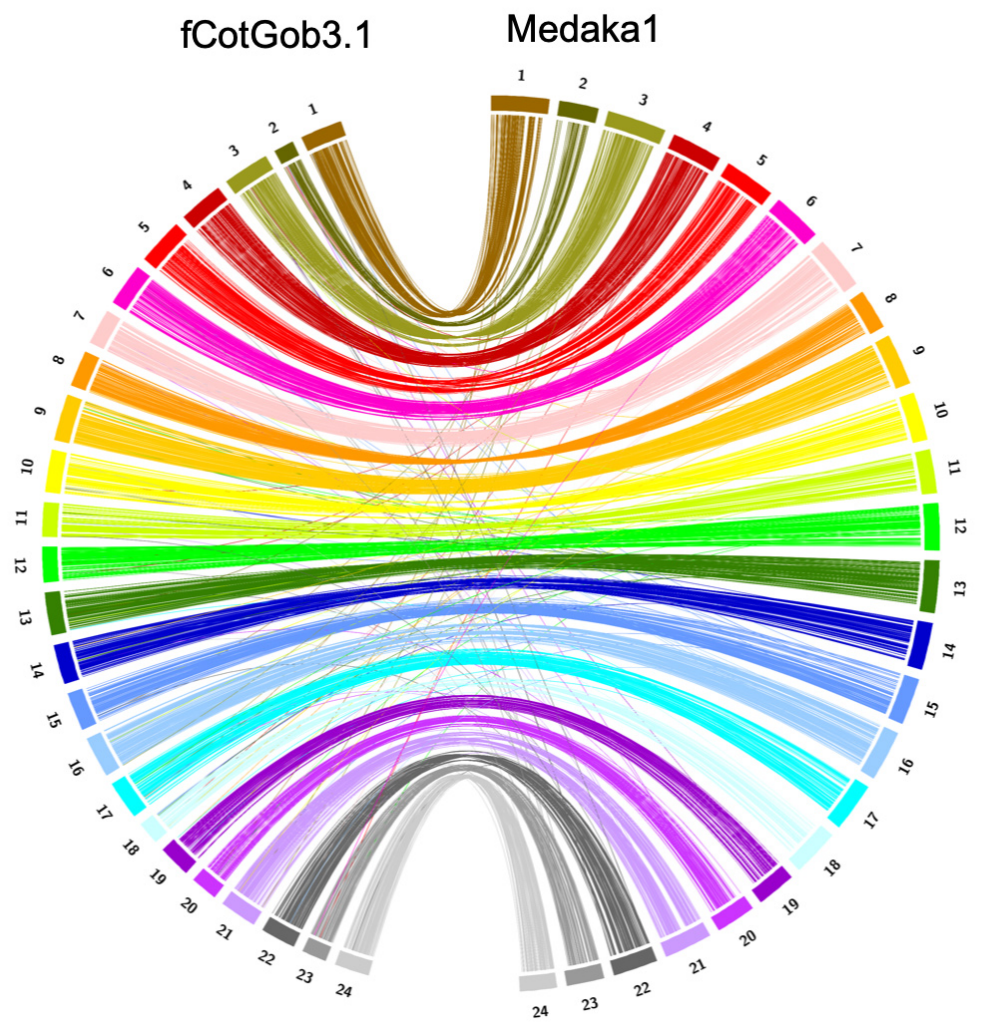
Syntenic relationships of fCotGob3.1 assembly with Japanese medaka HdrR chromosomes, based on single copy orthologs. Visualised in Circos ( Krzywinski *et al.,* 2009).

**Figure 4.**
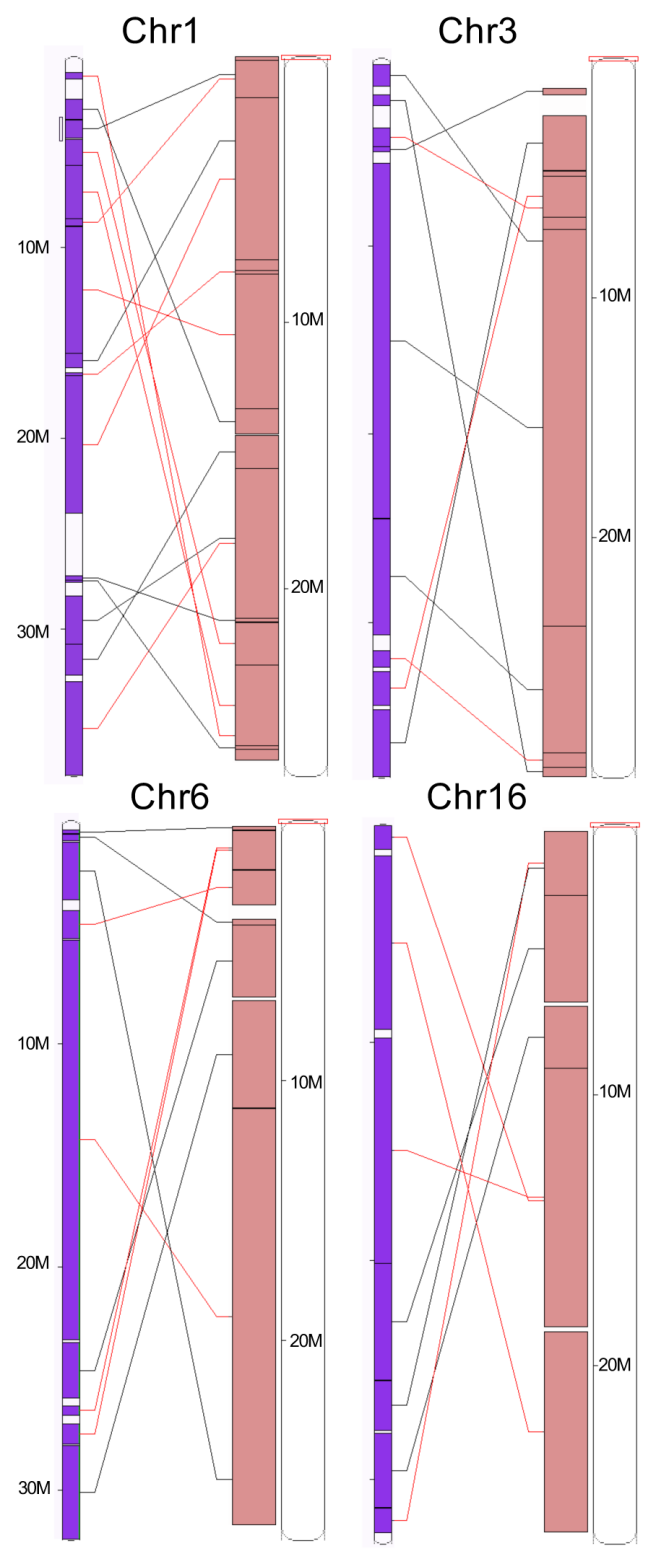
Examples of conserved synteny between Japanese medaka HdrR (purple) and fCotGob3.1 (pink) from chromosomes 1, 3, 6, and 16 (source: Ensembl).

The final assembly has a total length of 609 Mb, in 322 sequence scaffolds with a scaffold N50 of 25 Mb ( Figure 1; Table 1). The majority (94.36%) of the assembly sequence was assigned to 24 chromosomal-level scaffolds using the Hi-C data ( Figure 2; Table 2). The assembly has a BUSCO ( Simão *et al*., 2015) gene completeness score of 93.4% using the actinopterygii reference set (with -sp zebrafish parameter). The chromosomes clearly show a one-to-one relationship with those in the Japanese medaka ( *Oryzias latipes*) HdrR assembly GCA_002234675.1 ( Figure 3 and Figure 4), with 3671 of the 3780 complete and single copy BUSCO genes present in both genomes found on homologous chromosomes (97.1%), and were thus named correspondingly. Analysis of conserved syntenies detected no major interchromosomal rearrangements in the approximately 195 million years since the divergence of medaka and *C. gobio* lineages ( Steinke *et al*., 2006), but many intrachromosomal rearrangements ( Figure 4). While not fully phased, the assembly deposited represents one haplotype. Contigs corresponding to the second haplotype have also been deposited.

**Table 1.** Data information for *Cottoperca gobio*, fCotGob3.1 genome assembly.

Project accession information	
Assembly identifier	fCotGob3.1
Species	*Cottoperca gobio* ( *Cottoperca trigloides*)
Specimens	fCotGob3 (PacBio, 10XG and BioNano), fCotGob2 (Hi-C and RNA-seq)
	fCotGob1 (RNA-seq)
NCBI taxonomy ID	56716
BioProject	PRJEB30272
Study accession	PRJEB19273
BioSample IDs	SAMEA104132835 (fCotGob1) SAMEA5365137 (fCotGob1.brain1) SAMEA5365124 (fCotGob1.gonad1) SAMEA5365123 (fCotGob1.muscle1) SAMEA104242971 (fCotGob2) SAMEA4872137 (fCotGob2.spleen1) SAMEA104242975 (fCotGob3)
**Raw data accessions**	
Pacific Biosciences SEQUEL I	ERR2219167 - ERR2219176
10X Genomics Illumina	ERR2639757 - ERR2639760
Hi-C Illumina	ERR4179340 - ERR4179344
BioNano	ERZ1392783 - ERZ1392785
RNA-seq	ERR3132340 (fCotGob1.brain1) ERR3132342 (fCotGob1.gonad1) ERR3132341 (fCotGob1.muscle1) ERR2639616 (fCotGob2.spleen1)
**Genome assembly**	
Assembly accession	GCA_900634415.1
Accession of alternate haplotype	GCA_900634435.1
Span (Mb)	609
Number of contigs	766
Contig N50 length (Mb)	5,939,854
Number of scaffolds	322
Scaffold N50 length (Mb)	25,156,145
Longest scaffold (Mb)	30.48
BUSCO genome score	C:93.4%, [S:90.5%, D:2.9%], F:1.3%, M:5.3%, n:4584

**Table 2.** Chromosomal pseudomolecules in the genome assembly fCotGob3.1, of species *Cottoperca gobio* - GCA_900634415.1.

Name	INSDC	RefSeq	Size (Mb)	GC%	Protein	Gene
1	LR131916.1	NC_041355.1	27.06	40.8	1,808	1,175
2	LR131927.1	NC_041356.1	12.92	41.9	792	681
3	LR131933.1	NC_041357.1	30.03	40.3	1,487	919
4	LR131934.1	NC_041358.1	28.95	40.7	1,629	1,007
5	LR131935.1	NC_041359.1	30.48	40.9	2,033	1,302
6	LR131936.1	NC_041360.1	27.68	40.9	1,823	1,143
7	LR131937.1	NC_041361.1	23.07	41	1,619	1,088
8	LR131938.1	NC_041362.1	23.43	41.2	1,836	1,194
9	LR131939.1	NC_041363.1	30.07	41	1,888	1,158
10	LR131917.1	NC_041364.1	27.44	40.8	1,407	992
11	LR131918.1	NC_041365.1	22.19	40.8	1,440	909
12	LR131919.1	NC_041366.1	22.9	40.6	1,424	850
13	LR131920.1	NC_041367.1	27.74	41	1,542	1,029
14	LR131921.1	NC_041368.1	25.7	40.6	1,627	1,134
15	LR131922.1	NC_041369.1	24.96	41	1,365	967
16	LR131923.1	NC_041370.1	26.58	41	1,811	1,094
17	LR131924.1	NC_041371.1	25.16	40.8	1,663	1,228
18	LR131925.1	NC_041372.1	14.93	41.8	1,018	690
19	LR131926.1	NC_041373.1	21.06	41.2	1,563	969
20	LR131928.1	NC_041374.1	17.6	41.4	964	649
21	LR131929.1	NC_041375.1	24.1	40.6	1,400	937
22	LR131930.1	NC_041376.1	22.61	41.3	1,415	1,026
23	LR131931.1	NC_041377.1	15.93	41.9	973	594
24	LR131932.1	NC_041378.1	22.44	41.1	1,229	1,184
Unplaced	-	.	34.34	41.6	2,093	1,676

## Gene annotation

An Ensembl annotation was generated for the fCotGob3.1 assembly using RNA-seq data generated from 4 tissues (brain, muscle, ovary, and spleen). The annotation for assembly fCotGob3.1 was released in Ensembl under database version 99.31 ( Hunt *et al*., 2018) (for fish clade annotation information see 2019-09: fish clade gene annotation). The resulting Ensembl annotation includes 60,811 transcripts assigned to 21,662 coding and 2,823 non-coding genes ( Channel bull blenny - Ensembl). RefSeq annotation is also available as NCBI Cottoperca gobio Annotation Release 100 ( Table 2).

## Methods

### Specimen acquisition and nucleic acid extractions

Both specimens used to generate the genome assembly were collected south of the Falkland Islands/Islas Malvinas in 2004 (Lat Long: -52° 40’, -59° 12’) during the ICEFISH 2004 Cruise (International Collaborative Expedition to collect and study Fish Indigenous to Sub-Antarctic Habitats; led by H. W. Detrich ( Detrich *et al*., 2012)) of the *RVIB Nathaniel B. Palmer*. Following euthanasia, fresh blood was collected from specimen fCotGob3, and spleen tissue (used for Hi-C) was collected from specimen fCotGob2 and was flash frozen in liquid nitrogen. Blood was processed immediately, whereas flash frozen spleen was preserved in the -80 freezer until processing. For RNA sequencing, tissue samples from two specimens were used (fCotGob2 - spleen, and fCotGob1 - brain, skeletal muscle, ovary). The additional tissues (fCotGob1) were preserved in RNALater and kept frozen until extraction. The tissues were sampled by T. Desvignes, H. W. Detrich, and J. H. Postlethwait from a specimen captured northwest of the Falkland Islands in 2018 by the Falkland Islands Fisheries Department ( Grass *et al*., 2018).

High molecular weight (HMW) DNA from fresh blood cells was prepared using an agarose plug extraction protocol ( Smith *et al*., 2010). Blood DNA was initially stabilised in agarose plugs and then shipped to Sanger Institute where the final steps of the extraction were performed using a BioNano Tissue extraction protocol. Quality control (QC) of HMW DNA was performed using the Femto Pulse instrument (Agilent). Total RNA was extracted from approximately 20–40 mg of tissue, from brain, skeletal muscle, ovary and spleen tissues using the RNeasy Qiagen extraction kit (Qiagen). QC was performed using Qubit HS RNA kit, and Agilent Bioanalyzer Nano chips. Only extracts with RIN value >8 were used for sequencing.

### Sequencing

PacBio continuous long read (CLR) and 10X Genomics linked read sequencing libraries were constructed according to manufacturers’ instructions. Sequencing was performed by the Scientific Operations core at the Wellcome Sanger Institute on PacBio SEQUEL I and Illumina HiSeq X instruments. Hi-C data were generated using the Arima Hi-C kit v1 by Arima Genomics. BioNano data were generated on Saphyr (dual enzyme) at Bionano Genomics. RNA-seq was performed on HiSeq 4000 with 150bp insert paired end (PE) libraries.

### Genome assembly

An initial PacBio assembly was made using Falcon-unzip ( Chin *et al*., 2016) without repeat-masking during overlap detection with Dazzler. The contigs from this assembly were first scaffolded by comparing them to a second wtdbg ( Ruan & Li, 2019) assembly using cross_genome, then they were scaffolded further using the 10X data with scaff10X, and then with BioNano two-enzyme hybrid scaffolding using Solve v3.2.1. The original PacBio data were then used to fill gaps with PBJelly ( English *et al*., 2012) and polish with Arrow. The resulting assembly was then polished again using the 10X Illumina data, by mapping with bwa mem ( Li, 2013), calling variants with freebayes ( Garrison & Marth, 2012), and correcting homozygous non-reference variants with bcftools consensus. Contiguity was increased further by filling gaps with the contigs from a second wtdgb assembly, which was made using PacBio reads corrected with Canu ( Koren *et al*., 2017). This assembly was re-polished with Arrow and freebayes, and retained haplotigs were identified with Purge Haplotigs ( Roach *et al*., 2018). Finally, the assembly was scaffolded to chromosomes using Arima Hi-C data with Salsa ( Ghurye *et al*., 2017). The scaffolded assembly was checked for contamination and manually improved using gEVAL ( Chow *et al*., 2016). The manual curation included steps such as correcting mis-joins, improving concordance with all available data types, and Hi-C 2D map visualized in Juicebox to produce complete chromosomal units ( Durand *et al*., 2016). Curation resulted in 9 manual breaks, 114 manual joins and the removal of 102 regions representing false duplications, decreasing the scaffold count by 39% to 322 and increasing the scaffold N50 by 68% to 25.2 Mb. The chromosomal-level scaffolds were named based on conserved synteny to the medaka assembly ( *Oryzias latipes,* Assembly accession GCA_002234675.1). The genome was further analysed within the BlobToolKit environment ( Challis *et al*., 2020). Software tools and versions used for assembly are listed in Table 3.

**Table 3.** Software tools used for genome assembly.

Software tool	Version	Source
Falcon- unzip	falcon-2018.03.12- 04.00	( Chin *et al*., 2016)
wtdbg	1.1	( Ruan & Li, 2019)
cross_ genome	2014-08-22	https://sourceforge. net/projects/phusion2/files/ cross_genome/
PBJelly	PBSuite_15.8.24	( English *et al*., 2012)
Canu	1.6	( Koren *et al*., 2017)
Purge Haplotigs	v1	( Roach *et al*., 2018)
Juicebox		( Durand *et al*., 2016; Robinson *et al*., 2018)
scaff10x	1.0	https://github.com/wtsi- hpag/Scaff10X
Solve	Solve3.2.2_ 08222018	https://bionanogenomics. com/downloads/bionano- solve/
arrow	GenomicConsensus 2.2.2	https://github.com/ PacificBiosciences/ GenomicConsensus
Bwa-mem	0.7.17-r1188	( Li, 2013)
freebayes	v1.1.0-3-g961e5f3	( Garrison & Marth, 2012)
bcftools consensus	1.7	http://samtools.github. io/bcftools/bcftools.html

## Data availability

### Underlying data

European Nucleotide Archive: *Cottoperca gobio* (channel bull blenny) genome assembly, fCotGob3.1. BioProject accession number PRJEB30272; https://identifiers.org/ena.embl:PRJEB30272.

The *C. gobio* genome sequencing is part of the Wellcome Sanger Institute’s Vertebrate Sequencing project, and of the Vertebrate Genomes Project (VGP) ordinal references programme ( Rhie *et al*., 2020). All raw data and the assembly have been deposited in the ENA. Raw data and assembly accession identifiers are reported in Table 1.

## Reporting guidelines

Not applicable.

## Consent

Not applicable.

## Author contributions

RD, JHP, HWD, SAM, IB: designed the experiment. IB, MS, KO: generated data. HWD, TD, JHP: provided samples. SAM, IB, JW, ZN, RD: performed data analysis. JW, WC, KH, JT: performed data curation. VGP Consortium: provided guidance for methodology development. EAM, RD: supervised the work and provided funding. IB: wrote the manuscript. All authors reviewed and edited the final version of the manuscript.

## Author information

The Vertebrate Genomes Project Consortium includes Farooq O. Al-Ajli, Iliana Bista, Dave Burt, William Chow, Karen Clark, Hiram Clawson, Joanna Collins, Andrew J. Crawford, Joana Damas, Federica Di Palma, Mark Diekhans, Richard Durbin, Olivier Fedrigo, Paul Flicek, Giulio Formenti, Arkarachai Fungtammasan, Erik Garrison, Jay Ghurye, M. Thomas P. Gilbert, Jennifer Marshall Graves, Dengfeng Guan, Bettina Haase, Leanne Haggerty, Brett T. Hannigan, Robert S. Harris, Alex Hastie, David Haussler, Jinna Hoffman, Kevin Howe, Kerstin Howe, Erich D. Jarvis, Warren E. Johnson, Juwan Kim, Heebal Kim, Sarah B. Kingan, Byung June Ko, Klaus-Peter Koepfli, Sergey Koren, Jonas Korlach, Zev Kronenberg, Woori Kwak, Tanya M. Lama, Chul Lee, Joyce Lee, Harris Lewin, Kateryna D. Makova, Tomas Margues-Bonet, Fergal Martin, Patrick Masterson, Shane A. McCarthy, Paul Medvedev, Claudio V. Mello, Axel Meyer, Mark Mooney, Jacquelyn Mountcastle, Robert W. Murphy, Eugene W. Myers, Luis Nassar, Gavin J.P. Naylor, Zemin Ning, Stephen J. O’Brien, Sadye Paez, Benedict Paten, Sarah Pelan, Trevor Pesout, Adam M. Phillippy, Martin Pippel, Damon-Lee Pointon, Arang Rhie, Oliver A. Ryder, Simona Secomandi, Siddarth Selvaraj, Beth Shapiro, Maria Simbirsky, Ying Sims, Michelle Smith, Ivan Sovic, Emma C. Teeling, Constantina Theofanopoulou, Francoise Thibaud-Nissen, James Torrance, Alan Tracey, Marcela Uliano-Silva, Byrappa Venkatesh, Sonja C. Vernes, Brian P. Walenz, Tandy Warnow, Wesley C. Warren, Sylke Winkler, Jonathan Wood and Guojie Zhang.
