# Supplementary material for: The genome sequence of the channel bull blenny, Cottoperca gobio (Günther, 1861)
Source: Wellcome Open Res. 2020 Jun 24;5:148. [Version 1] doi: 10.12688/wellcomeopenres.16012.1 (PMC7649722; doi:10.12688/wellcomeopenres.16012.1)
Supplement: Peer review history

# Peer review: Reviewer response for version 1

**DOI:** 10.21956/wellcomeopenres.17562.r41211

**Published:** 2020-11-06

**Authors:** Chenhong Li, Liang Lu

**Affiliations:** 1 Shanghai Universities Key Laboratory of Marine Animal Taxonomy and Evolution, Shanghai Ocean University, Shanghai, China; 2 Shanghai Ocean University, Shanghai, China

The authors reported a well-assembled genome of an interesting species. The methods used are sound and sufficient, and the datasets are easily accessible. The only thing I found is that they maybe want to elaborate the importance of sequencing this species and to use it as an outgroup for comparative genomics in the Antarctic notothenioids, in addition to conservation of this species per se. Or, if possible make a comparison with the published Antarctic fish, which would increase the interest much more and maybe number of citations as well.

 Comments from my Doctoral student (not that I agree with them all):

1. Have the barcodes been trimmed when using 10XIllumina data for polishing?
2. The genes were annotated, but how their function was predicted was not clear.
3. The sample numbers, BioProject, BioSamples and the length of each chromosome can be lodged in the “data available” part, not in the main text.
4. The tools used were detailed in the text, which is redundant to include them in Table 3.
5. The biological background was touched in the introduction, but no any analyses were used to address any biological questions. (I understand that this is a data note though).

Are sufficient details of methods and materials provided to allow replication by others?

Yes

Is the rationale for creating the dataset(s) clearly described?

Partly

Are the datasets clearly presented in a useable and accessible format?

Yes

Are the protocols appropriate and is the work technically sound?

Yes

Reviewer Expertise:

Molecular phylogenetics, genomics, bioinformatics.

We confirm that we have read this submission and believe that we have an appropriate level of expertise to confirm that it is of an acceptable scientific standard.

# Peer review: Reviewer response for version 1

**DOI:** 10.21956/wellcomeopenres.17562.r39467

**Published:** 2020-08-05

**Authors:** Kevin Bilyk

**Affiliations:** 1 Department of Biology, Montclair State University, Montclair, NJ, USA

The manuscript is reporting the newly available genome assembly for *Cottoperca gobio*, a basal notothenioid that diverged before the evolution of the cold-adaptations and cold-specialization that define the cryonotothenioids. One of the major challenges in studying the Antarctic notothenioids has been limited availability of genetic and genomic information from phylogenetically close temperate species, making comparative investigations difficult. The *C. gobio* genome plays an incredibly important role filling this gap, providing a temperate companion to the recent *Eleginops maclovinus* genome. These provide a far more appropriate baseline to evaluate the changes that have come with evolution in a polar environment than the traditional model fish species all of which are far more phylogenetically distant. The genome sequencing, assembly, and annotation are all well described and appear handled appropriately. Personally, I can only say that I am happy that this genome is now available and look forward to utilizing it in my own work over the coming months.

Are sufficient details of methods and materials provided to allow replication by others?

Yes

Is the rationale for creating the dataset(s) clearly described?

Yes

Are the datasets clearly presented in a useable and accessible format?

Yes

Are the protocols appropriate and is the work technically sound?

Yes

Reviewer Expertise:

Comparative physiology, transcriptomics, genomics and bioinformatics.

I confirm that I have read this submission and believe that I have an appropriate level of expertise to confirm that it is of an acceptable scientific standard.
